# The Influence of an Enriched Environment in Enhancing Recognition Memory in Zebrafish (*Danio rerio*)

**DOI:** 10.3389/fvets.2021.749746

**Published:** 2021-11-12

**Authors:** Cairsty DePasquale, Nicole Kemerer, Nathan White, Monica Yost, Jordan Wolfkill, Jennifer Sturgill, X. Li

**Affiliations:** ^1^Department of Biology, Pennsylvania State University – Altoona, Altoona, PA, United States; ^2^Department of Mathematics and Statistics, Pennsylvania State University – Altoona, Altoona, PA, United States

**Keywords:** cognition, environmental enrichment, learning and memory, zebrafish (*Danio rerio*), object recognition

## Abstract

Environmental enrichment is used to increase social and physical stimulation for animals in captivity which can lead to enhanced cognition. Fundamental to the positive effect enrichment has on the brain is that it provides opportunities for captive animals to recognize and discriminate between different stimuli in the environment. In the wild, being able to discriminate between novel or familiar stimuli has implications for survival, for example finding food, hiding from predators, or even choosing a mate. The novel object recognition (NOR) test is a cognitive task that is used extensively in the rodent literature to assess object recognition and memory, where the amount of time an animal spends exploring a novel vs. familiar object is quantified. Enrichment has been shown to enhance object recognition in rodents. More recently, the use of the NOR test has been applied to another animal model, zebrafish (*Danio rerio*), however, the effects of enrichment have not yet been explored. In the current study we looked at the effects of enrichment on object recognition in zebrafish using the NOR test. Adult zebrafish were housed in either enriched conditions (gravel substrate, plastic plants, shelter, heater and a filter) or plain conditions (heater and filter only) for 6 months before behavioral NOR tests were conducted. Enriched fish showed a preference for a novel object over a familiar one at a distance but did not show a preference during close inspection. Control fish did not show a preference at either distance. Our results suggest that enrichment can enhance zebrafish ability to discriminate between novel and familiar objects, but distance from the object may be an important factor. Future research is needed to determine whether any enhancements in object recognition are a result of an increase in sensory stimulation from being reared with enrichment, or whether it is due to a reduction in stress reactivity.

## Introduction

Environmental enrichment is used extensively across many different animal taxa [e.g., fish: (1–3), birds: (4, 5), mammals: (6, 7)] to enhance physical and social stimulation in a captive setting. One aspect of enrichment that is fundamental to the benefits that it provides is the ability to recognize and discriminate between different stimuli in the environment. For example, animals react differently to a stimulus if it is novel vs. something familiar (8). In the wild, being able to discriminate between different novel or familiar stimuli has implications for survival, for example finding food, hiding from predators, or even choosing a mate (9).

Discriminating between different environmental stimuli requires that an individual assess if the stimulus is something they recognize from past encounters or not (10). The novel object recognition (NOR) test is a cognitive task that is used extensively in the rodent literature to assess object recognition and memory (11), where the amount of time an animal spends exploring a novel vs. familiar object is quantified and compared. Research on animal models of human disease, particularly rodents, has shown that they will spend more time exploring an object that is novel compared to an object that is familiar to them (11). More recently, the use of the NOR test has been applied to many non-mammalian animal models [for review see (12)].

The effects of environmental enrichment on performance in the NOR task has been explored in rodents, with most studies showing enhanced learning of novel objects by animals living with enrichment. For example, enrichment has been shown to increase the amount of time exploring a novel vs. familiar object in young (13, 14) and aged (15) rats compared to non-enriched conspecifics. Since there was no difference in total exploration rates between enriched and non-enriched groups, the results indicate that enhancement of memory could be attributed to learning alone and not just a difference in overall exploratory activity between the experimental groups. Rodents reared with more complexity in their environment (increased physical structures and objects to interact with) habituated faster to novel objects (8). Moreover, rats exposed to lifelong intermittent enrichment (3 1-h sessions a week for 18 months) showed enhanced learning in the NOR compared to non-enriched individuals (16). Different strains of rodent models used for various neurobehavioral disorders have also shown a similar effect of enrichment on increased exploration of a novel object, indicating enhanced learning (17–19). In contrast, Viola et al. (20) showed that young CF1 mice exposed to enrichment spent less time overall exploring objects than conspecifics not exposed to enrichment, however, enriched mice still spent more time exploring the novel vs. familiar object.

The NOR task has only recently been used to assess object recognition in zebrafish (21–24). Similar to rodent models, zebrafish show a propensity to explore novel objects over familiar objects (21, 23, 25) when the objects are simple geometric shapes such as spheres, however, this effect does not hold true with more complex or large objects (24). Moreover, it has been suggested that zebrafish are more sensitive to differences in color rather than shape or size of an object (22). Zebrafish color preference has been explored extensively with mixed results; a number of studies suggest that zebrafish are attracted to shorter wavelength colors such as blue and green and tend to avoid longer wavelength colors such as red and/or yellow (26–28), however, others have suggested they are attracted to red and green colors and avoid yellow and blue (22, 28–30). Although the effects of enrichment on novel object recognition have not yet been explored in zebrafish, several molecular pathways have been implicated in enhanced object recognition. For example, nicotine has been shown to enhance learning and memory in zebrafish subjected to the NOR test (21, 22) presumably through activation of acetylcholine receptors, which have been suggested to play a part in improved learning and memory in rodents (31). In addition, learning and memory in rodents has been shown to be inhibited by deacetylation of histones (32); zebrafish treated with phenylbutyrate (a drug that deactivates deacetylation of histones) exhibit improved learning in the NOR (22). Finally, the hormone 17β-estradiol which is known to be involved in modulating neural plasticity and neurogenesis, was shown to enhance novel object recognition in zebrafish (33).

In the current study we investigated the effects of enrichment on novel object recognition in zebrafish. We chose objects with simple, geometric shapes and colors that would not evoke strong responses either way (avoidance or attraction). We predicted that fish housed with enrichment would exhibit increased exploration of a novel object over a familiar one compared to control fish.

## Materials and Methods

### Experimental Set Up

**Figure 1:**
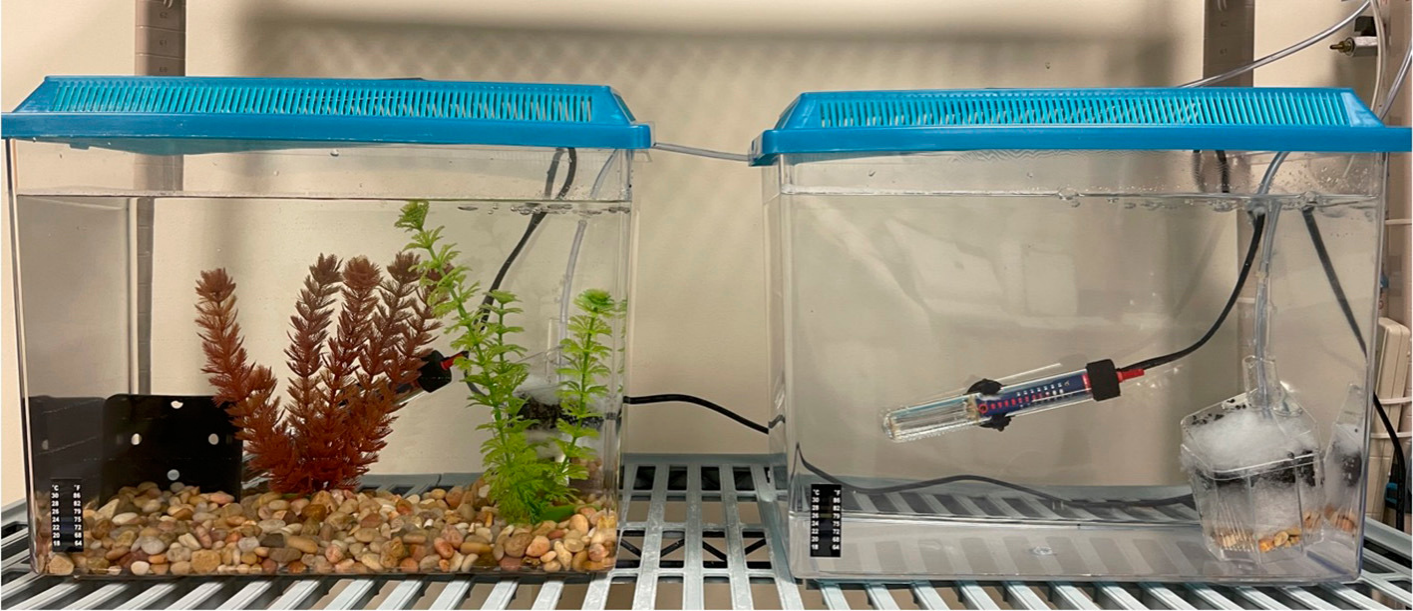
Design of the home tanks. Enriched on left, Control on right. Enriched tanks contained a biofilter, heater, gravel substrate, plastic shelter, and two plastic plants. Control tanks contained a biofilter and heater only.

One-year old wild-type zebrafish (*n* = 96) were randomly distributed across two experimental groups, enriched and control, with equal numbers of each sex in each tank (8 tanks of each treatment, 6 fish in each tank). Enriched tanks had a small triple-flow corner biofilter (Lee's Aquarium and Pet Products, USA; Model number: 13405), heater (Penn-Plax Cascade Heat Aquarium Heater, 50 W), gravel substrate (rinsed and dried, grade <1 cm, 2 cm deep), two plastic plants (Pet Solutions, USA; one green and one red, 14 cm tall) and a small plastic shelter (black plastic plant pot, 9 cm). The shelter and both plastic plants in all enriched tanks were moved around once a week during cleaning, however, the final location of these enrichment items was consistent across all enriched tanks (Figure 1). Control tanks had a biofilter and a heater only. All home tanks were length = 35 cm × width = 19 cm × height = 28 cm, with a water depth of 25 cm. Tanks were placed on two shelving units standing side by side, each with four shelves, two tanks per shelf (one enriched and one control). The fish were maintained on a 12 L: 12 D cycle with a water temperature of 25 ± 1°C. The fish were fed daily with commercial flake food (TetraMin® Tropical Flakes) and live cultures of brine shrimp (*Artemia* sp.). All experimental and husbandry procedures were approved by the Pennsylvania State University's Animal Care Committee (protocol 201800369).

### Novel Object Recognition Test

**Figure 2:**
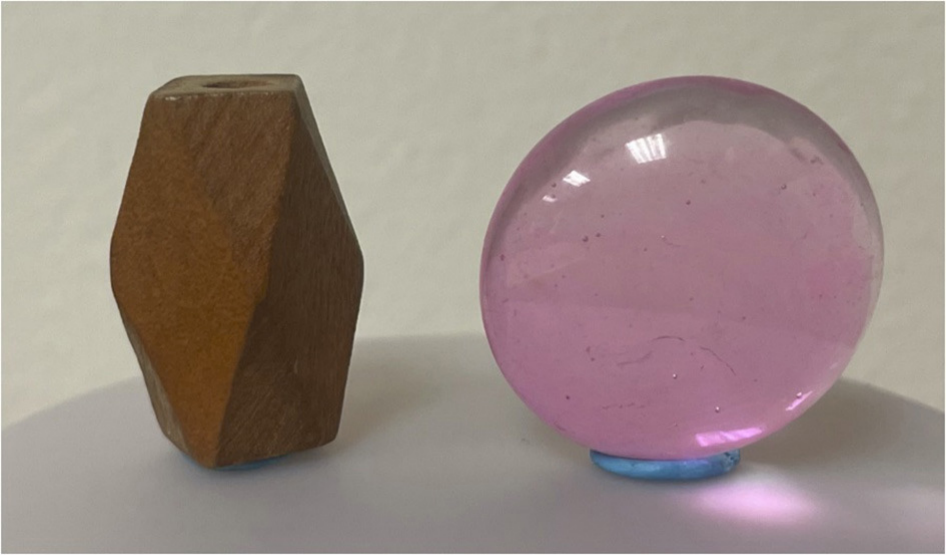
The two objects used in the NOR test; a simple spherical pink bead and a simple rod-shaped brown bead.

After 6 months of experimental conditions, all fish were tested in the NOR test. Six testing chambers were constructed using two large testing tanks (length = 76 cm, width = 76 cm, height = 30 cm) and dividing each into three equal sections (length = 25 cm, width = 25 cm) using gray non-transparent plexiglass dividers. A marker was used to place a black dot where each object would be placed (equidistant from all 3 walls of the chamber) to ensure the objects were being placed in the same location for each fish. Two different stimulus objects were used for NOR testing (Figure 2); a simple spherical pink bead (diameter = 2 cm) and a simple rod-shaped brown bead (diameter = 1 cm, height = 2 cm). Objects were pre-tested for saliency using non-experimental fish to ensure that individuals investigated both objects equally, thus indicating that the objects were equally interesting to zebrafish. The objects were attached (with a small amount of blue sticky tack) to the bottom of each experimental chamber. The role (familiar or novel) of the two stimulus objects was counterbalanced and psuedorandomly exchanged for each fish so that equal numbers of both enriched and control fish received the brown bead vs. the pink bead as the novel object. The testing tank was filled with sump water to a depth of 18 cm that was kept at within the same temperature range as the experimental home tanks.

**Figure 3:**
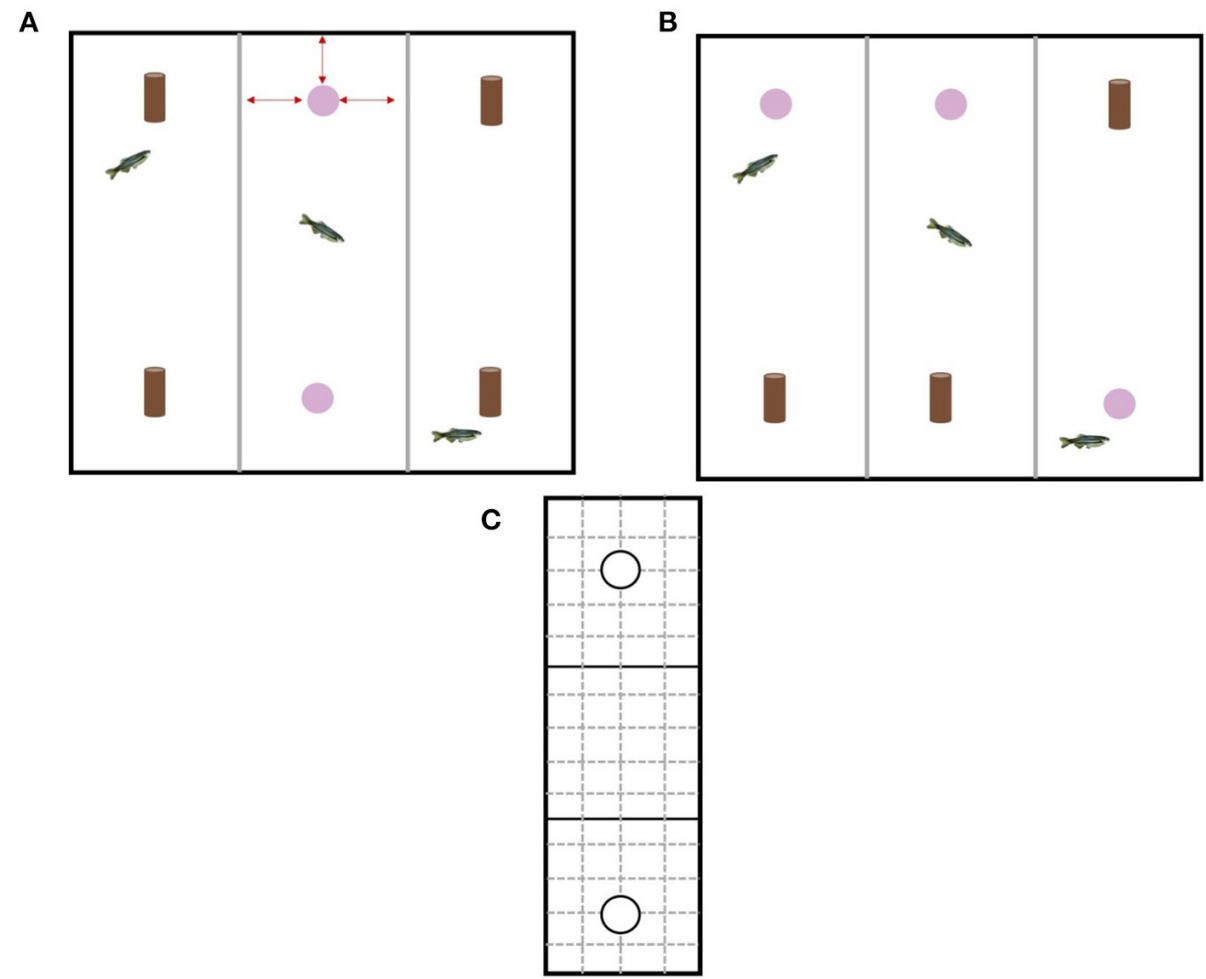
**(A)** Design of the experimental tank during the acquisition phase. The experimental tank had three different chambers for individual testing of fish. Gray opaque plastic dividers separated each chamber. During the acquisition phase each fish received two copies of the same object, however, which type of object (pink bead or brown bead) was alternated to provide equal numbers of fish within each treatment that had been exposed to the different objects. Red arrows indicate the object was equidistant from the three walls surrounding it **(B)** Design of the experimental tank during the retention phase. During the retention phase each fish received one copy of the original (familiar) object and one copy of the new (novel) object. The location of the object was rotated to minimize any effects of side bias. **(C)** Schematic of the acetate used to analyze the behavioral videos. Solid black lines depict the three different zones (object 1 zone, neutral middle zone, object 2 zone) and solid black circles depict the encounter zone around each object. Solid black lines and dashed gray lines indicate lines used for quantifying movement rate.

Over the course of 4 days, each fish was given 3 h to acclimatize to the testing chamber without any stimulus objects prior to testing. Each fish from all experimental home tanks was individually netted from their home tank and placed carefully in a testing chamber (1 fish per chamber). We chose 3 h because acclimatization of zebrafish to the testing apparatus in previous studies using the NOR have ranged from 5 min (24) to 24 h (23). In addition, 3 h is a sufficient period of time for any potential stress hormones from handling to decrease to baseline levels (34). The test tank was replaced with new sump water prior to testing starting to eliminate the effects of stress hormones that may have been released into the water by the fish during acclimatization. Once all fish had been acclimatized, testing started. The NOR test was split into two phases: an acquisition phase and a retention phase (Figures 3A,B). The acquisition phase involved exposing each individual fish to two identical objects within each chamber for 10 min. The objects were already present in the tank when the fish was placed into the chamber. Fish were quickly (<30 s) and carefully netted from their home tank (if enrichment was present it was removed to make it easier to net the fish) and was placed in the center of the experimental chamber (to reduce any bias related to the fish being introduced closer to one of the objects). Once the last fish was placed in the 3rd chamber, timing started. The experimenter was careful to minimize any disturbances to fish already in a testing chamber by approaching the tank quickly and quietly. After the 10 min was over the objects were removed and the fish stayed in their individual testing chambers for a further 4 h (retention interval). A retention interval of 4 h was chosen because it has been shown that zebrafish are able discriminate between familiar and novel objects after a retention interval of only 2 h and as long as 24 h (23). Immediately following the 4 h retention interval, the retention phase started with the experimenter carefully netting the fish out the tank and placing in a holding tank so that each chamber could be set up for the retention phase. The retention phase involved exposing each individual fish to one familiar object and one novel object within each chamber for 10 min. The relative position of the two stimulus objects (top of chamber or bottom of chamber) was counterbalanced and pseudorandomly exchanged for each fish to reduce any side biases. Again, the fish was carefully and quickly netted from their holding tank and placed in the center of the experimental chamber. Timing started once the last fish was placed in the 3rd chamber. During both phases the experimenter left the room to minimize any outside disturbances and the fish were free to explore the objects and the chamber. Once the retention phase was over, the fish were removed from the test tank and placed in new experimental home tanks separate from fish still to be tested. The water in the test tanks was replaced with new sump water before stimulus objects and new fish were placed in each chamber. Video cameras secured to the ceiling were used to record fish behavior during the acquisition and retention phases. The videos were then analyzed using BORIS software (35). All video analysis was carried out by the same experimenter so as to reduce any experimenter bias. Acetate and a marker were used to divide each experimental chamber into a grid with three equal zones (object 1 zone, neutral middle zone, object 2 zone; 25 cm) and an encounter area (radius = 1 body length) around each object (Figure 3C). Variables collected included total time spent encountering object, total time in object zone, and movement rate (number of grid lines crossed/min).

### Statistical Analysis

Due to the position of the video camera, the sex of the zebrafish was not defined during statistical analysis because the identification of males and females was not reliable from the videos. Firstly, a linear model was used to determine if there were any differences between Enriched and Control fish in overall levels of exploration during the acquisition phase. The independent variables were treatment (Enriched or Control) and object type (pink ball or brown rod), and the dependent variable was movement rate. Linear models were then used to determine if there were any differences between Enriched and Control fish in the time spent encountering either object or entering either object zone during the acquisition phase. The independent variables that were included in the models were treatment (Enriched or Control), object type (pink or brown), and object location (top or bottom).

To determine if there was a difference between Enriched and Control fish in their preference to explore the novel object over the familiar one during the retention phase, a discrimination ratio was calculated. The discrimination ratio was used to analyze preference for one zone over the other as well as preference for approaching one object over the other and was expressed as:

Time spent exploring novel object/(Time spent exploring familiar object + time spent exploring novel object).

Where time spent exploring was either time in zone or time spent encountering. A discrimination ratio of 0.5 indicated no preference for one object over the comparison object. Discrimination ratios higher than 0.5 indicated a relative preference for the novel object, and scores lower than 0.5 indicated a relative aversion to the novel object.

Again, a linear model was used to determine if there were any differences between Enriched and Control fish in overall exploration during the retention phase. The independent variables were treatment (Enriched or Control), object type (pink ball or brown rod), novel object location (top or bottom), and object novelty (novel or familiar). The dependent variables were time spent encountering the object, time spent inside the object zone, or movement rate. To determine if there was a difference between Enriched and Control fish in their preference to explore a novel vs. familiar object during the retention phase, generalized linear models were conducted using the quasibinomial model in R. The discrimination ratio for time to encounter the object and time inside the object zone were used as the dependent variables. The independent variables were treatment (Enriched or Control), object type (pink ball or brown rod), and novel object location (top or bottom).

Interaction effects were excluded from the models if they were not significant. Fish were excluded from analyses if they did not move and remained frozen during the entire acquisition phase or retention phase (Enriched, *n* = 7; Control, *n* = 5). During one session the cameras failed to record, so those fish were excluded from the analyses (Enriched, *n* = 3; Control, *n* = 3). Thus, the final number of fish from each treatment included in the analyses was Enriched = 38 and Control = 41. Four fish from the Enriched treatment and two fish from the Control treatment did not encounter either the novel or familiar object during the retention phase, therefore these fish were excluded from these analyses only. All data were checked for normality using Q-Q plots of residuals. All analyses were performed using R (36) and significance was tested at α = 0.05. Values are quoted as mean ± s.e.m.

## Results

**Figure 4:**
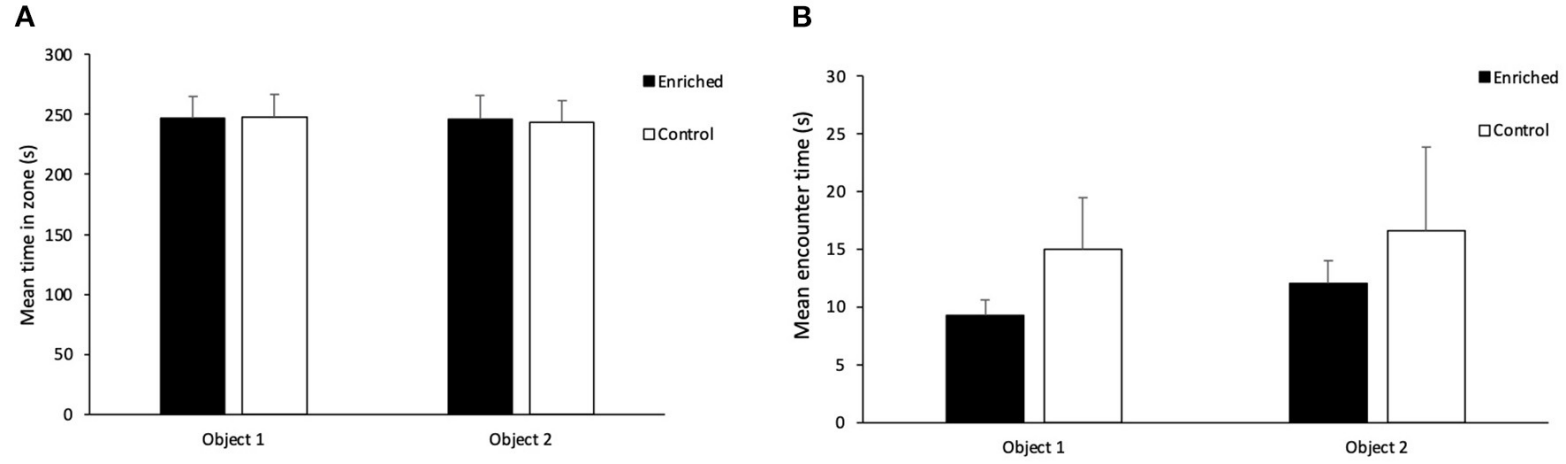
Acquisition Phase on the NOR. **(A)** Mean time in each object zone for Enriched and Control fish and **(B)** Mean encounter time for each object for Enriched and Control fish during the acquisition phase of the NOR. Values are displayed as mean ± s.e.m.

In terms of overall exploration during the acquisition phase, there was no effect of treatment (*t*_1_ = 0.08, *p* = 0.94) or object type (*t*_1_ = 1.05, *p* = 0.30) on movement rate. There was no effect of treatment (*t*_1_ = 0.02, *p* = 0.98; Figure 4A), object type (*t*_1_ = 0.80, *p* = 0.43) or object location (*t*_1_ = −0.11, *p* = 0.91) on the amount of time fish spent inside each zone during the acquisition phase. There was also no effect of treatment (*t*_1_ = -1.07, *p* = 0.28; Figure 4B), object type (*t*_1_ = -1.81, *p* = 0.07) or object location (*t*_1_ = 0.48, *p* = 0.63) on the time spent encountering the objects during the acquisition phase.

In terms of overall exploration during the retention phase, there was no effect of treatment in the time spent encountering the objects (*t*_1_ = −0.20, *p* = 0.84), time spent inside each zone (*t*_1_ = −0.08, *p* = 0.93), or movement rate (*t*_1_ = −0.66, *p* = 0.51). There was no effect of object type (*t*_1_ = 1.44, *p* = 0.15) or novel object location (*t*_1_ = 0.91, *p* = 0.36) on time spent encountering the objects. However, there was an interaction effect; fish spent more time encountering the pink ball novel object if it was in the top chamber of the experimental tank, but the opposite was true if the novel object was the brown rod (*t*_1_ = 2.05, *p* = 0.04; Supplementary Figure 1). There was no effect of object type or novel object location on time spent inside each zone (type: *t*_1_ = 0.62, *p* = 0.54; location: *t*_1_ = −2.04, *p* = 0.06), or movement rate (type: *t*_1_ = 1.12, *p* = 0.27; location: *t*_1_ = −1.77, *p* = 0.08). Finally, there was no effect of novelty on time spent encountering the objects (*t*_1_ = 1.07, *p* = 0.29), but time spent inside each zone was marginally significant (*t*_1_ = 1.95, *p* = 0.05); regardless of treatment, object type or location, fish spent more time in the novel object zone than the familiar zone (in seconds; novel: 293.57 ± 11.84; familiar: 257.82 ± 14.09).

**Figure 5:**
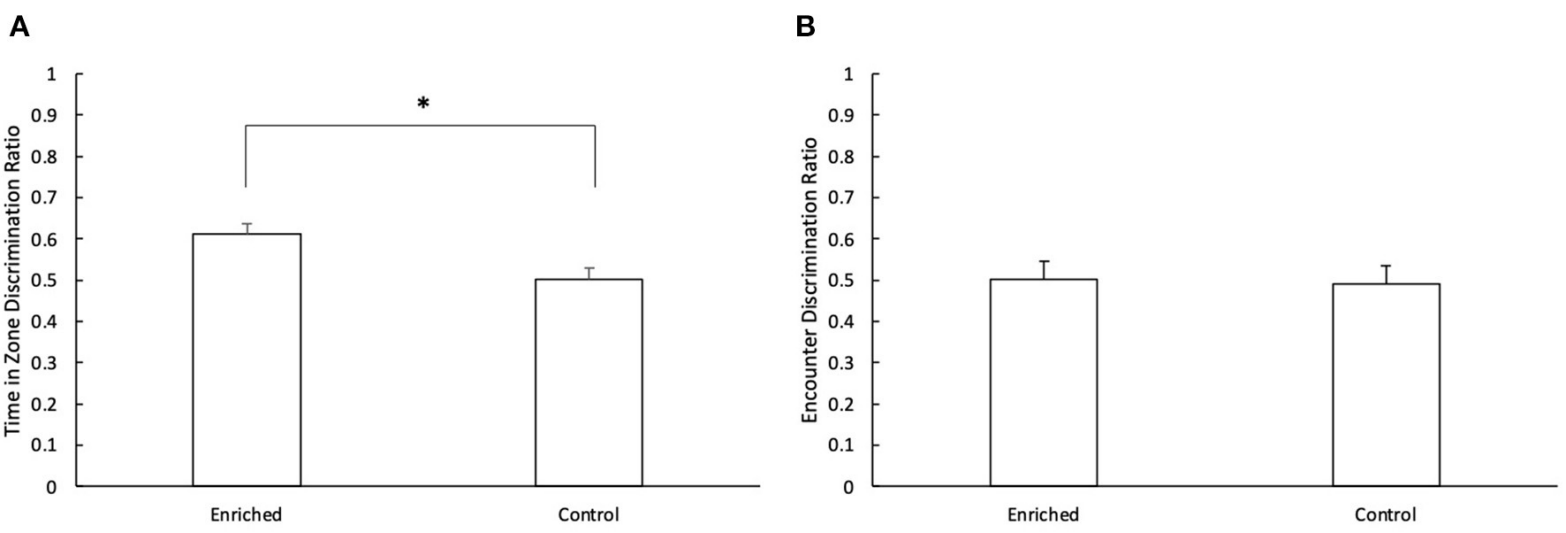
Retention Phase of the NOR. **(A)** Comparison of the time in zone discrimination ratio for Enriched and Control fish and **(B)** Comparison of the encounter discrimination ratio for Enriched and Control fish during the retention phase of the NOR. ^*^Denotes significant difference at P < 0.05. Values are displayed as mean ± s.e.m.

During the retention phase, there was a significant difference in the discrimination ratio of enriched and control fish for time in novel zone vs. familiar zone (*t*_1_ = 3.04, *p* < 0.01; Figure 5A). Fish reared in enriched conditions spent more time in the novel object zone during the retention phase than those reared in control conditions (Enriched: 0.61 ± 0.03; Control: 0.50 ± 0.03). There was no effect of object type (*t*_1_ = 0.81, *p* = 0.42) or object location (*t*_1_ = 1.52, *p* = 0.13) on the time spent inside each zone. There was no effect of treatment (*t*_1_ = −0.48, *p* = 0.63; Figure 5B), object type (*t*_1_ = 0.50, *p* = 0.62) or object location (*t*_1_ = 0.39, *p* = 0.70) on the discrimination ratio for time spent encountering the different objects.

## Discussion

To our knowledge, this is the first study to look at the effects of enrichment on novel object recognition in zebrafish. The results show that zebrafish reared with enrichment were better able to discriminate between a novel vs. familiar object from a distance compared to zebrafish reared in control conditions. Interestingly, neither Enriched or Control fish were able to discriminate between the familiar or novel object on close inspection when encountering the objects. Therefore, the results from the current study are in partial agreement with our predictions and with previous studies conducted on rodents.

During the acquisition phase, there were no differences in overall exploration between Enriched and Control fish, and no differences in the amount of time fish spent exploring the objects, either close up or from a distance. Thus, both groups interacted with the two identical objects for a similar amount of time. When we look at the discrimination ratio for time spent inside each zone during the retention phase, Control fish did not exhibit a preference for the novel object over the familiar one. In comparison, Enriched fish had an average discrimination ratio of 0.61, suggesting increased motivation and/or interest to explore the novel object vs. the familiar object compared to Control fish. Interestingly, there was no effect of treatment during the retention phase when we looked at the absolute levels of exploration in each zone. The inconsistent results can be explained by the different statistical methods used (linear regression vs. a generalized linear model) and the number of independent variables used. A discrimination ratio is typically used as the standard measure of recognition memory across different NOR studies, including those on zebrafish (21, 22, 24, 25) because it is more resistant to individual differences in exploration (37). Thus, we consider the discrimination ratio a more reliable measure. Similar studies on rodents have also shown that enrichment has a positive effect on object recognition memory [(38–41) but see]. It was suggested that mice reared with enrichment have a higher propensity to explore novel objects because they have been exposed to a more challenging environment (39). In zebrafish, enrichment has been shown to enhance other forms of learning and memory, such as spatial learning (42–44). Moreover, enrichment has been shown to increase neurogenesis (growth of new neurons) and neural plasticity in a number of different animals, including fish (2, 45). Therefore, in the current study zebrafish reared with enrichment may have exhibited enhanced object recognition memory because they previously experienced more stimulating environmental conditions, and this had a positive effect on cognition. A number of studies have reported beneficial effects of enrichment on cognition in juvenile fish raised with enrichment during a critical period of development (2, 42, 46). In this study we investigated the effects of 6 months of enriched conditions on adult zebrafish, however, it would be interesting to know if Enriched fish would have shown a stronger response had the fish been raised in the different conditions from a younger age.

Stress has been shown to influence exploratory behavior in zebrafish, with many studies reporting a decrease in exploratory behavior in response to different stressors, including net chasing and social isolation (42, 47, 48). The NOR paradigm used in the current study potentially exposed subjects to isolation stress, not only during behavioral testing in the acquisition and retention phase, but also during the 4-h retention interval and the 3 h acclimatization period before testing started. Moreover, although we tried to minimize handling before and during testing, all fish had to be transferred from their home tanks to the testing chamber before the acquisition phase and were placed in a holding tank to allow new objects to be placed in the chamber before the retention phase, both of which could have exposed the fish to further stress. Past research on zebrafish has shown that enrichment can decrease the stress response (49) and decrease anxiety-like behavior (42), leading to a better ability to cope with stress. Enrichment has also been shown to reduce the fear response in fish, making them less likely to exhibit neophobic behaviors (50). Thus, even though fish from both treatments were exposed to the same handling procedures and social isolation during testing, Enriched fish may have been able to cope with these stressors and the testing environment more effectively than Control fish, allowing them to perform better.

Contrary to our predictions, we did not see any differences between Enriched and Control fish, nor did either treatment group show any preference toward encountering either object as exhibited by their discrimination ratios (Enriched: 0.50 ± 0.04; Control: 0.49 ± 0.04). The fact that we see a higher discrimination ratio for Enriched vs. Control fish in terms of the time spent in the novel zone compared to the familiar one, but we see no difference in the discrimination ratio for close encounters between the two treatment groups is evidence that close inspection was not needed for Enriched fish to gain enough information to identify whether the object was novel or not. It has been suggested that object recognition at a distance is possible in an aquatic setting, where sensory information can be received through the lateral line system or through detection of water movement (12). On the other hand, the lack of discrimination by both treatment groups when encountering the objects could be due to the relatively small amount of time spent encountering objects during the 10 min trial (in seconds; Enriched: 21.22 ± 3.16; Control: 22.29 ± 2.75). Moreover, we excluded six fish across both treatment groups that did not even enter either encounter zone during the retention phase. It has been reported that the amount of exploration during the NOR should be representative of normal exploratory behavior and allow for a meaningful statistical interpretation (37). We chose 1 body length (~3 cm) as the size for the encounter zone which is similar to the 3.6 cm used in Luccon Xiccato and Dadda (Luccon Xiccato and Dadda, 2014), however, other NOR studies using zebrafish have used larger encounter zones of 8–10 cm (21, 22, 24, 25). Thus, the size of the encounter zone in the current study may have limited the amount of exploration we detected and thus not been a true representation of close inspection.

There was no influence of object type (brown rod or pink ball) on exploration during the acquisition phase, suggesting that both objects were equal with respect to motivation for exploration. Furthermore, there was no effect of object type on overall exploration in each zone, or on the discrimination ratios for either time spent encountering the objects or time in each zone. However, zebrafish did spend more time encountering the pink ball novel object if it was in the top chamber of the experimental tank, but the opposite was true if the novel object was the brown rod. Zebrafish have a propensity to exhibit color preference, however, the exact order of those preferences is still debated (28). Moreover, it has been documented that objects of a novel color and/or shape are known to increase exploration in zebrafish, but size does not induce such a response (22, 26). In the current study, the type of object and its location were counterbalanced among subjects, so our experimental design should have controlled for any bias effects. We also chose objects that were not known to have ethological significance for the zebrafish; we picked simple, geometric objects of the same size with neutral colors—brown and light pink and a ball and a rod of the same size. Similar objects (a small pink sphere made of glass and a yellow plastic hexagonal-shaped prism) have been used in a previous study on object recognition in zebrafish (23). However, in the current study there may have been subtle differences in the appearance of the objects from different angles depending on the lighting in the room that were only apparent on close inspection. For example, the shiny surface of the pink ball may have been more distinctive under a certain light compared to the matte surface of the brown rod. In addition, the limited size of the encounter area around each object, and thus the relatively short encounter times across all fish, could have made any small random difference in time spent during close inspection appear more significant.

In conclusion, the results of the current study show that enrichment can improve the ability of zebrafish to discriminate between a novel and a familiar object at a distance, however, any effect of enrichment on object recognition during close inspection was inconclusive. We investigated the effects of 6 months of enriched housing conditions on adult (1 year-old) zebrafish. It would be interesting to know if the results of the current study would have been different if the zebrafish had been raised in different housing conditions. Future research should take into account how the size of the area around the object is defined to quantify close inspection of the objects, as well as any differences in the appearance of the objects in the testing arena. Furthermore, more research is needed to determine whether any enhancements in object recognition are a result of an increase in sensory stimulation from being reared with enrichment, or whether it is due to a reduction in stress reactivity. Physiological and behavioral measures of stress, as well as neurological tools could help to answer these questions.

## Data Availability Statement

The raw data supporting the conclusions of this article will be made available by the authors, without undue reservation.

## Ethics Statement

The animal study was reviewed and approved by Penn State University IACUC (Protocol 201800369).

## Author Contributions

CD conceived the original idea and supervised the project. NK, NW, MY, and JW conducted behavioral experiments. JS analyzed the videos. XL conducted the statistical analyses. CD wrote the manuscript with support from NK, NW, MY, JW, and JS. All authors contributed to the article and approved the submitted version.

## Funding

This work was supported by the Pennsylvania State University.

## Conflict of Interest

The authors declare that the research was conducted in the absence of any commercial or financial relationships that could be construed as a potential conflict of interest.

## Publisher's Note

All claims expressed in this article are solely those of the authors and do not necessarily represent those of their affiliated organizations, or those of the publisher, the editors and the reviewers. Any product that may be evaluated in this article, or claim that may be made by its manufacturer, is not guaranteed or endorsed by the publisher.
