# Supplementary material for: The Influence of an Enriched Environment in Enhancing Recognition Memory in Zebrafish (Danio rerio)
Source: Front Vet Sci. 2021 Nov 12;8:749746. doi: 10.3389/fvets.2021.749746 (PMC8632956; doi:10.3389/fvets.2021.749746)
Supplement: Supplementary file 1 [file Data_Sheet_1.DOCX]

Supplemental Figure 1. Interaction of novel object type (brown rod or pink ball) and novel object location (bottom or top) on mean encounter time during the retention phase. Values are quoted as mean ± s.e.m.
